# Low Impact Velocity Modeling of 3D Printed Spatially Graded Elastomeric Lattices

**DOI:** 10.3390/polym14214780

**Published:** 2022-11-07

**Authors:** Jose Angel Diosdado-De la Peña, Charles M. Dwyer, David Krzeminski, Eric MacDonald, Alberto Saldaña-Robles, Pedro Cortes, Kyosung Choo

**Affiliations:** 1 Material Science and Engineering, Youngstown State University, Youngstown, OH 44555, USA; 2 Advanced Manufacturing Research Center, Youngstown State University, Youngstown, OH 44555, USA; 3 EOS North America, Pflugerville, TX 78660, USA; 4 College of Engineering, University of Texas at El Paso, El Paso, TX 79968, USA; 5 Department of Agricultural Mechanical Engineering, University of Guanajuato, Irapuato 36500, Guanajuato, Mexico; 6 Mechanical Engineering, Youngstown State University, Youngstown, OH 44555, USA

**Keywords:** additive manufacturing, spatially graded lattices, finite element analysis, thermoplastic polyurethane

## Abstract

Additive manufacturing technologies have facilitated the construction of intricate geometries, which otherwise would be an extenuating task to accomplish by using traditional processes. Particularly, this work addresses the manufacturing, testing, and modeling of thermoplastic polyurethane (TPU) lattices. Here, a discussion of different unit cells found in the literature is presented, along with the based materials used by other authors and the tests performed in diverse studies, from which a necessity to improve the dynamic modeling of polymeric lattices was identified. This research focused on the experimental and numerical analysis of elastomeric lattices under quasi-static and dynamic compressive loads, using a Kelvin unit cell to design and build non-graded and spatially side-graded lattices. The base material behavior was fitted to an Ogden 3rd-order hyperelastic material model and used as input for the numerical work through finite element analysis (FEA). The quasi-static and impact loading FEA results from the lattices showed a good agreement with the experimental data, and by using the validated simulation methodology, additional special cases were simulated and compared. Finally, the information extracted from FEA allowed for a comparison of the performance of the lattice configurations considered herein.

## 1. Introduction

In recent years, the interest in additive manufacturing (AM) has been growing across the world since it enabled the fabrication of structures through a successive layer-upon-layer deposition process, allowing the creation of intricate geometries that are difficult to build using traditional manufacturing technologies [1,2]. The usage of AM technologies has also facilitated the construction of features at several hierarchical scales, with customized material processing in different points or layers, and has opened up a wide range of configurable designs to be explored [3]. These characteristics have made AM a suitable option for the fabrication of lattice structures, which are a type of cellular structure, along with foams and honeycombs. Lattices have many outstanding properties over the latter, such as lightweight and higher strength, energy absorption, and vibration reduction, which have been extensively studied [4]. Lattice structures have been utilized for different industrial applications, such as scaffolds for tissue and bone replacement, automotive and aerospace parts for noise isolation and weight reduction, as well as on diverse protective applications [5].

AM has allowed the study of lattice structures made from diverse polymeric, metallic, and ceramic materials through the different methodologies defined by the International Organization for Standardization (ISO) and the American Society for Testing and Materials (ASTM) [6]. For instance, metallic lattice research has widely focused on Ti-6Al-4V, AlSi10Mg, and 316L stainless steel through electron beam melting (EBM) and selective laser melting (SLM) [4,5,7]; conversely, polymeric lattices studies have mostly concentrated on polylactic acid (PLA), acrylonitrile butadiene styrene (ABS), and nylon-based powders by using selective laser sintering (SLS) and fused deposition modeling (FDM) [4,5,7,8]. For instance, ABS lattices have also been fabricated via stereolithography (SLA) with photo-polymeric resin [9], and thermoplastic elastomer (TPE) has been printed via SLS [10].

The capabilities of AM technologies have also allowed various researchers to build lattices with elevated levels of complexity. Indeed, several works have investigated enclosed truss-based unit cell geometries based on cubic, diamond, triangular, pyramidal, body-centered cubic (BBC), dodecahedron, octahedral, tetrahedron, and tetrakaidecahedron or Kelvin cell [5,7,9,10,11,12,13,14,15,16,17,18,19,20,21,22,23,24,25,26,27,28], among many other geometries. Surface-based geometries and triply periodic minimal surface (TPMS) configurations as a spherical shell, gyroid, IWP, OCTO, Schwarz, TPMS diamond, and TPMS primitive have also been studied [5,7,14,15,29,30,31,32]. Likewise, modifications and combinations of the basic truss-based geometries can also be found in the literature, such as the re-entrant dodecahedron, reinforced BCC, octet, and face-body-centered cubic (FBCC), as well as structures with varying distribution of the volume fraction of each unit cell in the 3D design domain, also known as spatially graded lattices [5,7,9,18,22,24,25,26,27,33,34,35,36,37,38,39,40]. The manufacturability of some of the aforementioned geometries could be limited by the post-processing method, such as heat treatment, curing, or cleaning [10].

Lattice structures are mainly evaluated by their stiffness and energy absorption capabilities through quasi-static compression tests [9,10,11,12,13,14,16,17,19,22,27,30,34,35,36,37,38,39,41] and dynamic compression tests [18,22,23,25,35,42]. The behavior of the lattices is mainly affected by the geometry of its unit cell, the relative density, and the properties of the parent material [4,28,43,44]; therefore, multiple tests are carried out on the base (solid) material to characterize its mechanical behavior under tensile, flexural, and compressive loads [8,11,13,14,19,27,29,31,34,35,36,38,39,42,45,46], commonly via universal testing machines (UTM). The mechanical characterization of the base material and the appropriate adjustment to a material model enables the improvement of the design process and the study of lattice structures. For instance, Altamimi et al. [41] investigated thirty different periodic lattice structures based on a cubic crystal structure initially via finite element analysis (FEA), by fitting the behavior of black PA1102 polyamide to an elastic-perfectly plastic constitutive material model and implementing a quasi-static compression simulation. Based on the predicted effective elastic properties of yield and buckling strength, a reduced number of cases were selected, fabricated (through SLS), and tested, where the experimental results validated the numerical results with good agreement.

The dynamic compression analysis of lattice structures can be performed through a variety of experimental tests, including the split Hopkinson’s pressure bar (SHPB) and the drop impact test, as well as analytical and numerical methods, where current developments in simulation software are removing some of the limitations faced in the foregoing studies that led to predict absorption behavior only by quasi-static compression loading tests [23]. Such was the case of Ling et al. [35], who used two different polymer resins to create three different octet-truss lattice structures by SLA and performed drop tests with a Rosand impactor tester. In this work, they restricted their simulations only to quasi-static compressions as they were not able to set a failure criterion. On the other hand, Fadeel et al. [18] fitted the mechanical behavior of ABS parts to a plasticity material model with ductile and shear damage failure criteria. This work considered the simulation of four variations in BBC lattices for the ABS core of sandwich structures under low-velocity impact. The models showed a good agreement with a previous experimental work [47], where lattices were manufactured through FDM.

Although the published research on general-purpose dynamic models mimicking the compressive behavior of polymeric lattice configurations is still limited [18], important contributions have been generated by several research works. For instance, Eren et al. [25] printed variations in the octet, diagonal, and BCC lattices using a translucent acrylic-based photopolymer via PolyJet 3D printing, which was compressed under a controlled elevated strain rate. The FEA of the lattices assumed a bilinear isotropic hardening material model and boundary conditions similar to those used in the experimental setup. Here, similar stress–strain curves were obtained for numerical and experimental works, although post-yielding was slightly different for the octet and BCC unit cells. Habib et al. [46] built octagonal, Kelvin, and honeycomb lattices with a polyamide thermoplastic (PA12) through multi-jet fusion (MJF), which were tested with an SHPB at high strain rates. The authors fitted the base material behavior to the Johnson–Cook plasticity model and reproduced the experiment via FEA. The validated numerical results allowed the authors to evaluate vertically graded versions of the lattices and predict improvements to the energy absorption behavior. Jhou et al. [23] modeled a falling mass shock absorption test on BCC and edged-centered cubic (ECC) lattices employing FEA and used a linear isotropic elastic material model for thermoplastic polyurethane (TPU). The lattices were built using FDM and tested to verify the numerical results; the authors concluded that the structure deformation and peak acceleration were overestimated under the impact loading, and the use of non-linear hyperelastic material models might improve the numerical prediction. Clough et al. [42] studied elastomeric tetrahedral microlattices fabricated from mixtures of thiol-ene and urethane acrylate monomers through a collimated UV source and performed a double anvil dynamic compression test. This work also included FEA in the test, where the behavior of the lattice material was fitted to the neo-Hookean hyperelastic material model. Montgomery et al. [22] used a two-stage curing resin to manufacture uniform and functionally graded foams (FGF) using a non-symmetric Kelvin unit cell through grayscale digital light processing (g-DLP). A drop tester was used on these lattices, and the experimental results were reproduced via FEA, where a linear viscoelastic Maxwell model in combination with the Prony series was used to model the solid lattice material.

As it can be observed, a considerable number of studies on lattice structures have been investigated, mainly focusing on quasi-static loading conditions with a limited number of works under dynamic conditions and a lack of methodology description. Hence, there is a need for a modeling study that can provide a full methodological description of lattice structures under dynamic conditions, including the incorporation of spatially graded configurations at different orientations. Therefore, this work proposes the study of elastomeric lattices under quasi-static and dynamic compressive loads, along with numerical analysis through FEA. A Kelvin unit cell was selected to design and build non-graded and spatially graded lattice configurations. A commercially available TPU was chosen as the base material in order to induce high strain levels on the structure while avoiding its failure. Here, tensile test samples of the parent material were manufactured via SLS and later tested under quasi-static loading, while the lattices were assessed under both quasi-static and dynamic conditions. The mechanical characterization of the base material was used to fit the observed behavior to a non-linear hyperelastic material model, which was later used as the input for the numerical simulation of the different tests. The agreement between the experimental and numerical results has been used to validate the simulation methodology adopted, and this has allowed the special cases and applications of the material and graded configurations here investigated to be addressed.

## 2. Materials and Methods

This section discusses the experimental setups used for the quasi-static and dynamic compression tests, as well as the features and configuration of the numerical simulation through FEA.

### 2.1. Parent Material

EOS^®^ TPE 300, a TPU flexible material with 92 shore A hardness [48], was used as a base material and printed in an EOS^®^ SLS P770 printer using the default laser parameters and a 120-micron layer thickness. Five tensile test samples were printed from TPU powder according to Type IV ASTM D638 Standard [49]. All samples were printed in an x–y orientation (with the thickness in z-orientation), and the post-processing included a manual cleaning. One sample was stretched until rupture to record the maximum extension required for this material, reaching over 300% of the original length. The remaining samples were tested at 25 mm/min until rupture using an Instron 5500 R UTM, and the average stress–strain curve was calculated.

### 2.2. Lattices

**Figure 1:**
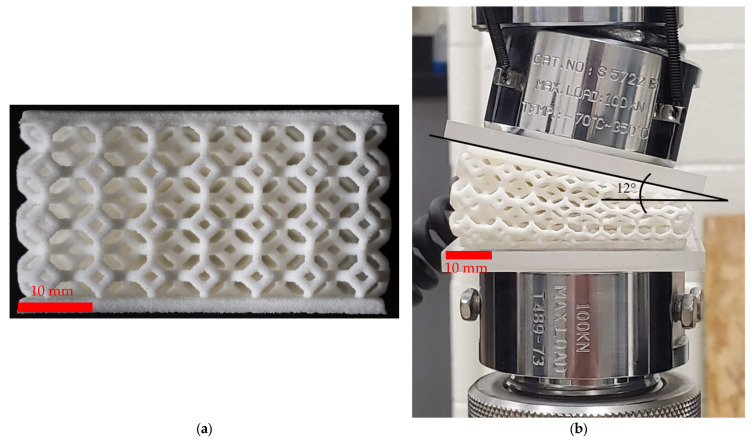
Printed Kelvin lattices: (**a**) non-graded as-built and (**b**) side-graded under quasi-static compression.

A Kelvin cell was selected as the lattice unit cell. This cell is also known as tetrakaidecahedron for closed cell or bitruncated cubic honeycomb for open cell cases [50,51]. Here, two configurations were investigated: (1) Non-graded lattice samples that had a uniform strut thickness across the entire volume; (2) Side-graded lattice samples that were based on a spatially varying strut thickness changing linearly through the width of the lattice from half-thickness struts to full-thickness struts. The design of each lattice was performed with a program called Mithril^®^ by Siemens^®^ and their transformative design (TRADES) program. The base material and printing settings were the same as for the tensile test samples. The size of the lattice structures was 50 × 50 × 25 mm with a lattice cell repetition of 7.1 mm in x, y, and z directions. Figure 1 shows the lattices here investigated.

#### Compression Testing

A quasi-static compression test on the non-graded lattice followed the ASMT D1621 standard [52] in an Instron 5500 R UTM, and the average stress–strain curve of at least three samples was calculated. The side-graded samples generated a 12° angle on the spherical seating compression platen of the UTM during the compression test, see Figure 1b; thus, the stress–strain curve for this lattice configuration was not here used.

**Figure 2:**
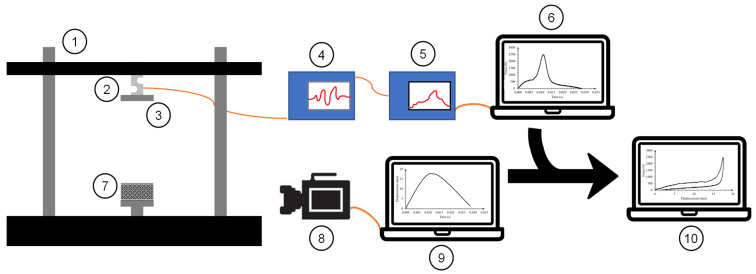
Scheme and data procedure for dynamic compression tests: (1) Drop tower; (2) Load cell; (3) Impactor; (4) Amplifier; (5) Oscilloscope; (6) Force-time data from the load cell; (7) Lattice sample; (8) High-speed camera; (9) Displacement-time data from high-speed video; and (10) Force-displacement compiled data.

Low-velocity impact testing was conducted on each of the samples of the different configurations. An impact tower outfitted with a 4.66 kg weight and a load cell was used to gather data on each impact. A drop height of 50 mm was used on each tested sample, where the impact lasted for up to 50 ms. About 10,000 data points were recorded on each test using a GW Instek^®^ oscilloscope and a Kistler^®^ charge amplifier connected to the load cell. The data from the load cell were matched up numerically with a 10,000 fps high-speed video clip from an Olympus i-Speed 3 camera [53]. An open-source software called Tracker [54] was used to calculate the displacement, velocity, and acceleration from the video clip based on the height of the sample and the frame rate. The data were then analyzed in a spreadsheet using the relationship between the mass, impactor height, and acceleration due to gravity as given by the classical potential energy equation: E = mgh. The force and displacement data were then compiled together to create force–displacement curves. The schematics of the impact test and the analysis procedure are shown in Figure 2. Particularly, the side-graded samples required the bottom plate of the impactor to be set to the 12° angle observed during the quasi-static compression in order to properly compare the lattice configuration performance.

### 2.3. Finite Element Analysis

A brief description of the multiple numerical models via FEA are shown herein, which were performed using ANSYS^®^ Mechanical and Workbench LS-DYNA^®^ suites. Geometries for each lattice case were built in Solidworks^®^ 2020 and imported into ANSYS^®^; details on the boundary and loading conditions, as well as materials settings, are described in the following sections.

#### 2.3.1. Tensile Test Modeling

The geometry of the test sample was built in a Design Modeler, ANSYS^®^ native CAD suite, and imported into ANSYS^®^ Mechanical. Boundary conditions similar to the experimental test were applied, where one end was fixed, and the other end was subjected to a displacement load to impose a normal strain of ε = 3.0. Here, the SOLID186 was selected as the element type.

It is worth mentioning that the TPU material here tested displays a rubbery behavior. Often, rubber-like materials are modeled by fitting experimental data to Arruba–Boyce, Ogden, Mooney–Rivlin, Neo-Hooke, and Yeoh hyperelastic material models, to name a few [55]. Each material model available in the literature is suitable for a particular material and experimental data set. For instance, the Mooney–Rivlin material model is appropriate to simulate natural rubber of up to a 100% tensile strain [56], while the Ogden 3rd-order material model better fits carbon black-reinforced rubber with tensile data of up to ε = 6.0, while the Yeoh material model works properly for natural rubber reinforced by carbon-black [57,58]. Particularly for TPU, Ogden, Yeoh, and Mooney–Rivlin material models can be fitted properly if diverse experimental data are within reach [59,60,61], but if only uniaxial tensile data are available, the Ogden model provides better results than the other two [59,62,63]. Therefore, in this work, the quasi-static average stress–strain curve was fitted to an Ogden 3rd-order material model by using HYPERFIT^®^ 2.181, which was used as the model input.

#### 2.3.2. Lattice Quasi-Static Compression

**Figure 3:**
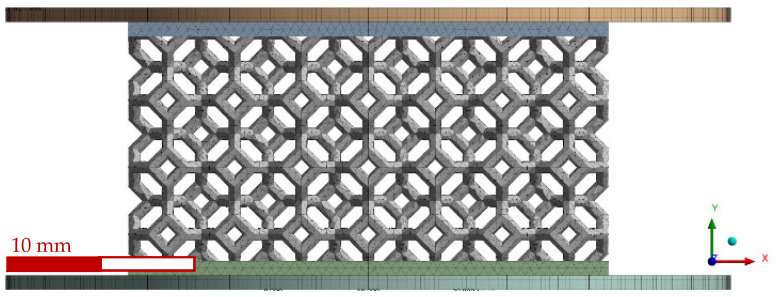
None-graded lattice full mesh with bottom plate and impactor.

Figure 3 shows the full representation of the mesh considered for the non-graded geometry, which includes a bottom plate, the lattice itself, and an impactor. The bottom plate was fully restricted, and the impactor moved only in the y-direction; both were modeled as rigid bodies with structural steel material properties (E = 210 GPa, ρ = 7850 kg/m^3^, υ = 0.3). Lattice material was also modeled with the Ogden 3rd-order material model, but SOLID285 was selected as the element type since it handles better incompressibility cases with non-linear materials [64]. The impactor was displaced downward 18.26 mm to impose over 60% compression. Frictional contacts were defined between the plates and the lattice with static and dynamic friction coefficients of 0.2 and 0.05, respectively; a similar contact was considered between the lattice struts with coefficients of 1.5 and 1.0 for the static and dynamic coefficients, respectively [65].

#### 2.3.3. Lattice Impact Loading

Non-graded geometry under impact loading was considered for the same conditions as those described for the quasi-static compression model. A mass point was added to the impactor to match the 4.66 kg of the actual impactor, and standard earth gravity was considered. Displacement on the impactor was substituted by an initial velocity of 1.1 m/s, which was assumed as the impact velocity from a 50 mm drop height. Here, the element type was changed to SOLID164 since it is the element available in ANSYS^®^ for explicit dynamic analyses.

##### Side-Graded Lattice

**Figure 4:**
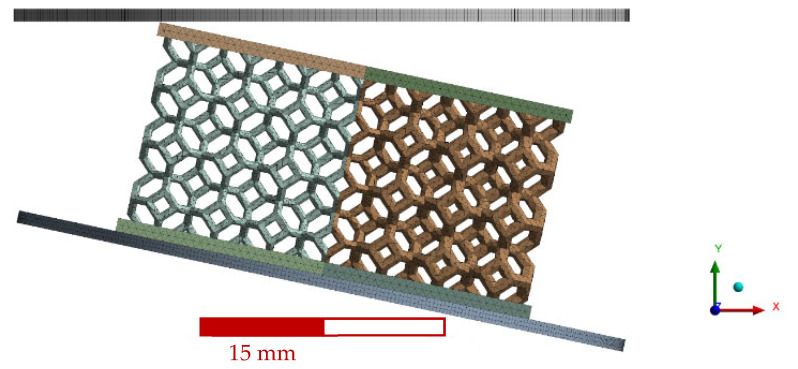
Side-graded lattice full mesh with bottom plate and impactor.

For this case, the boundary and loading conditions considered were similar to the simple impact conditions described for the non-graded lattice, with a 12° inclination difference. Figure 4 shows the full representation of the mesh for this case, including the bottom plate, lattice, and impactor.

##### Special Cases

Two additional cases were considered assuming modifications to each lattice. The first special case was similar to the simple impact on the non-graded lattice but with the 12° inclination used in the side-graded model in order to induce an oblique impact on the non-graded lattice. The second special case was the side-graded lattice inverted, where the impactor touched the coarse section first in the upper position of the geometry. These special cases were only numerically analyzed; no experiments were conducted with the described configurations.

##### Average Deceleration

Deceleration on the impactor for each impact case described was assessed by the average acceleration:

(1) $$\bar{a}=\frac{1}{t_{2}-t_{1}}\int_{t_{1}}^{t_{2}}a\left(t\right)dt,$$

Calculations from Equation (1) can also be used to determine the injury threshold similar to the head injury criterion (HIC) [66]; here, it was used to judge how fast the lattice decelerating on the impactor was proposed as the evaluation parameter for the lattices.

In this work, the acceleration histories can be extracted from the four impact simulations: the non-graded simple impact, non-graded oblique impact, side-graded, and inversed side-graded. By using Equation (1) on each acceleration history and assuming $t_{2}-t_{1}$ as the contact time, the average deceleration was calculated.

## 3. Results and Discussion

This section shows the experimental and numerical results obtained from the quasi-static and dynamic compression tests on the TPU tensile test samples and lattices.

### 3.1. Parent Material

**Figure 5:**
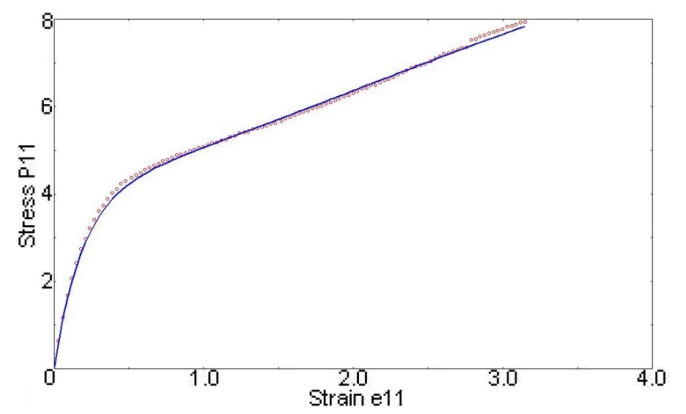
Ogden 3rd-order material model fitted to average experimental data.

The average stress–strain curve of the tensile test samples was calculated, and it is shown in Figure 5, where the Ogden 3rd-order material model is also displayed with the fitted parameters listed in Table 1.

**Figure 6:**
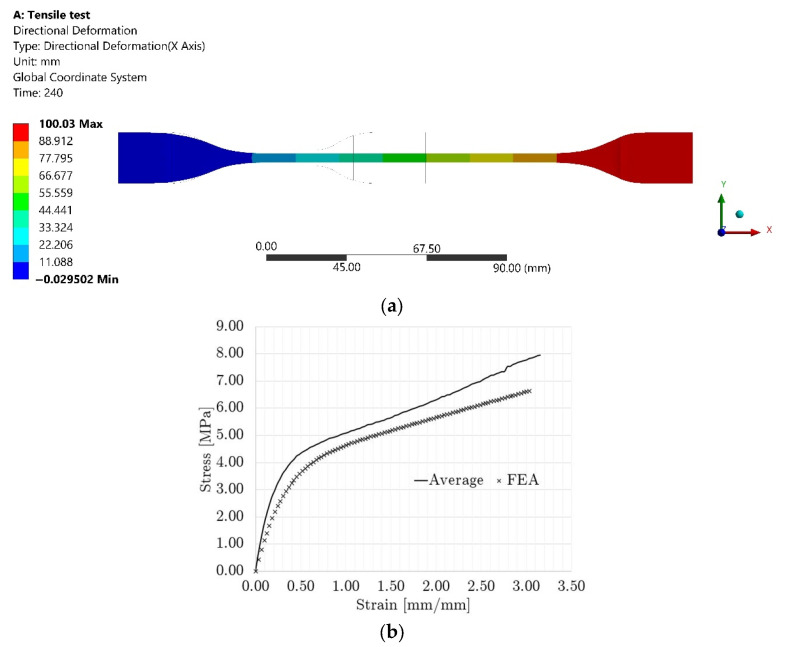
Modeling results for tensile test: (**a**) Final displacement and (**b**) Stress–strain curve comparison.

Figure 6a shows the final displacement result of the tensile test model, and Figure 6b shows the stress–strain curve calculated from the reactions and displacements at the boundaries of the model, where a similar profile and values can be observed. The base material results and the modeling of the tensile test proved that the Ogden 3rd-order material model represented properly the behavior of the parent material, EOS^®^ TPE 300.

It is worth mentioning that two main effects are reported in the literature for the TPU material: loading softening and strain-rate hardening. Loading and unloading TPU, either for the bulk material or lattice, produces stress softening after the first loading cycle, followed by a slight hysteresis, which stabilizes after the fourth loading cycle [67,68,69,70,71,72,73,74,75]. On the other hand, TPU is strain rate sensitive, and as it increases, the TPU transitions from rubbery to leathery to glassy behavior [70,76,77]. These effects are expected on the printed lattices and were included by tuning the average stress–strain curve by a factor for each case (quasi-static and impact), as implemented in other studies [61,78,79]. Thus, the parent material average stress–strain curve was factored, and the parameters were refitted for the quasi-static compression and impact modeling. The updated parameters are listed in Table 2 and were used in the simulations of each lattice.

### 3.2. Lattice Quasi-Static Compression

**Figure 7:**
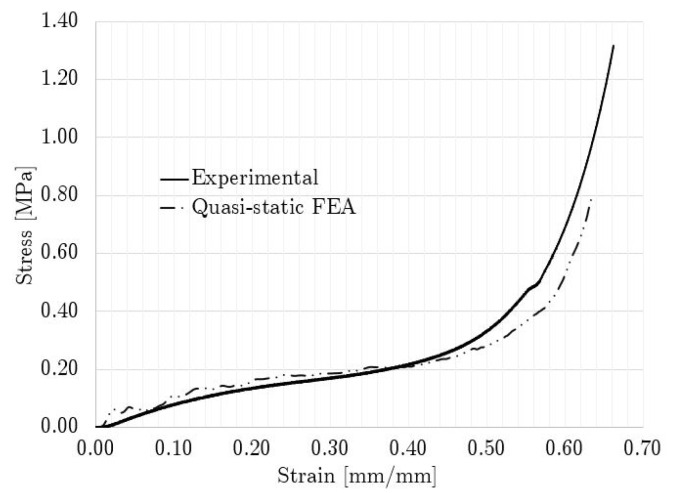
Quasi-static stress–strain curve comparison for non-graded lattice.

**Figure 8:**
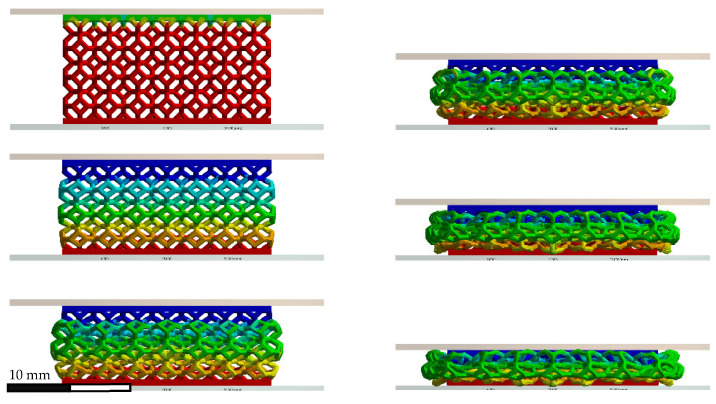
Quasi-static compression sequence for non-graded lattice.

Figure 7 shows a comparison between the experimental and numerical stress–strain curves for the non-graded lattice. The numerical stress–strain curve was calculated from the reactions and displacements at the boundaries of the model. The FEA compression sequence is shown in Figure 8.

The similarity of the quasi-static compression curves displayed in Figure 7 was verified by the FEA model and the fitting of the factored material model parameters. The discrepancies between the experimental and numerical results could be attributed to the assumptions on the boundary conditions. For instance, the numerical work assumed ideal conditions with friction coefficients from the literature, while the printed lattices might have had geometrical deviations, which could have led to local discrepancies in the friction conditions. Nonetheless, by integrating both curves in Figure 7, the calculated absorbed energy at 0.635 mm/mm differed only by 7% between the experimental and numerical results, which validated the non-graded quasi-static compression model.

### 3.3. Lattice Impact Loading

**Figure 9:**
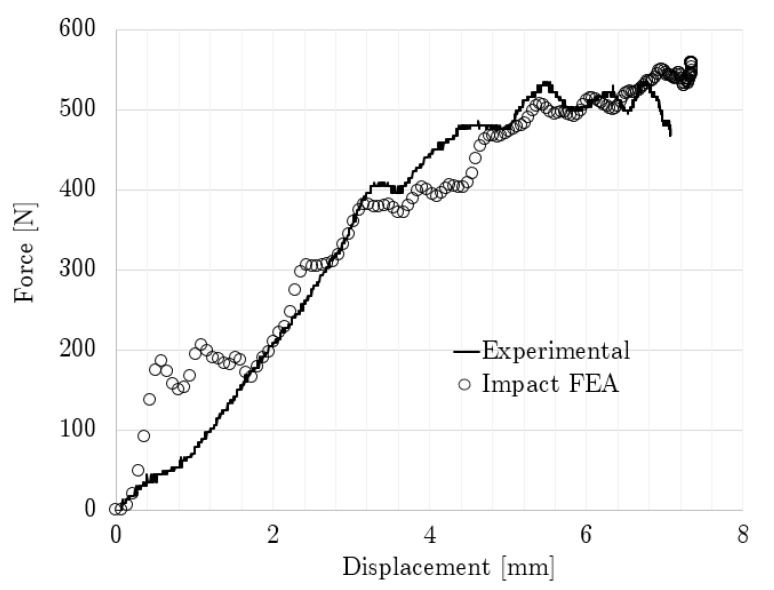
Impact force–displacement curve comparison for non-graded lattice.

**Figure 10:**
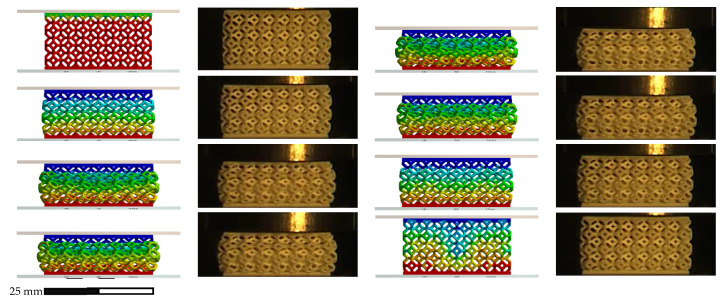
Compression sequence for non-graded lattice under simple impact.

Figure 9 shows the modeled and experimental force–displacement relationships up to the maximum compression reached by the non-graded lattice with simple impact. The compression sequence for this case is displayed in Figure 10, which contains both experimental and modeling results.

The non-graded simple impact time was calculated at 26.4 ms, while the experimental results indicated that the contact ended at 30 ms. By integrating Figure 9, the absorbed energy differed only by 2% at 0.267 mm/mm between the experimental and numerical results. From Figure 9, it can also be observed that the maximum displacement was 7.07 mm and 7.35 mm for the experimental and numerical results, respectively, while the maximum experimental force was 534.85 N and the numerical force was 558.84 N.

In the quasi-static and simple impact tests on the non-graded cases, a peak was observed at the beginning of the contact in Figure 7 and Figure 9; this behavior was similar to that reported by Heiml et al. [63]. This peak could be attributed to the numeric inelastic buckling that the structure had to overcome in order to collapse [80], and this was intensified during the impact testing due to the reduced time in which this effect had to take place. Here, the differences between the experimental and numerical stress–strain curves agree with the conclusion that the deformation energy is slightly lower in FEA than in the experiments [81].

**Figure 11:**
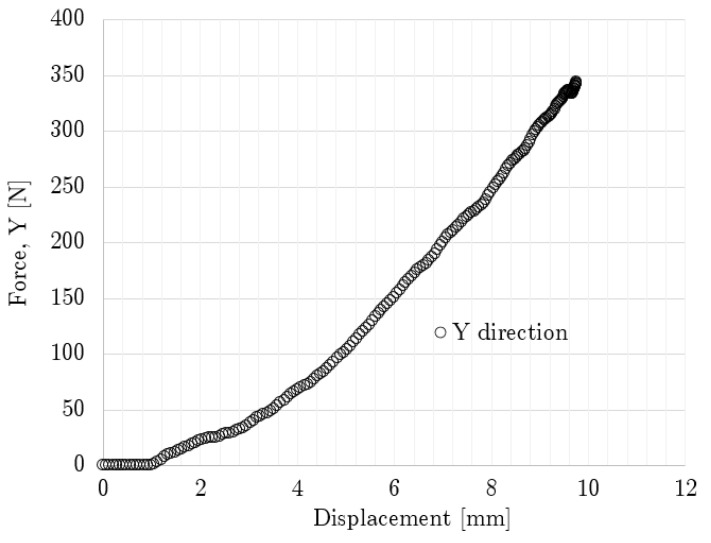
Impact force–displacement curve for oblique none-graded lattice.

The force–displacement curve obtained from the non-graded oblique impact case up to the maximum compression point is shown in Figure 11, where the results differed from those shown by the simple impact case. Here, the oblique case resulted in a higher maximum displacement (9.49 mm vs. 7.35 mm), but its peak force in the vertical direction was lower by almost 200 N, in comparison with the simple impact case, and the contact time was nearer to 30 ms. Only numerical results are included since this configuration was not experimentally tested.

**Figure 12:**
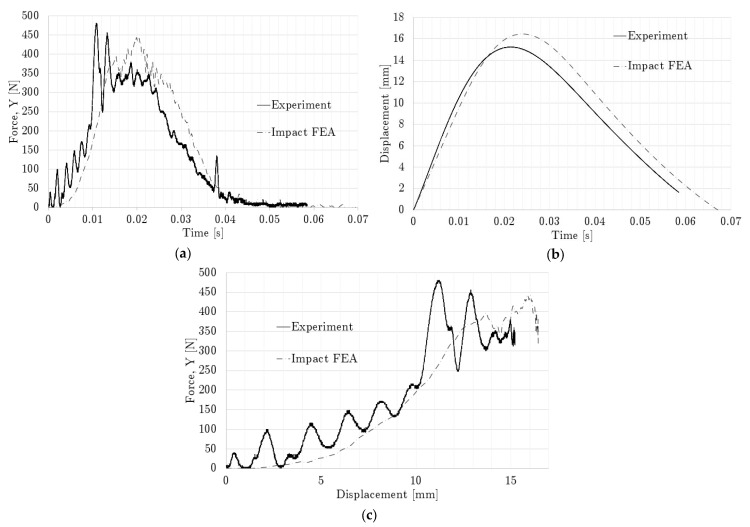
Impact loading comparison for side-graded lattice: (**a**) Force history, (**b**) Displacement history, and (**c**) Force–displacement curve.

**Figure 13:**
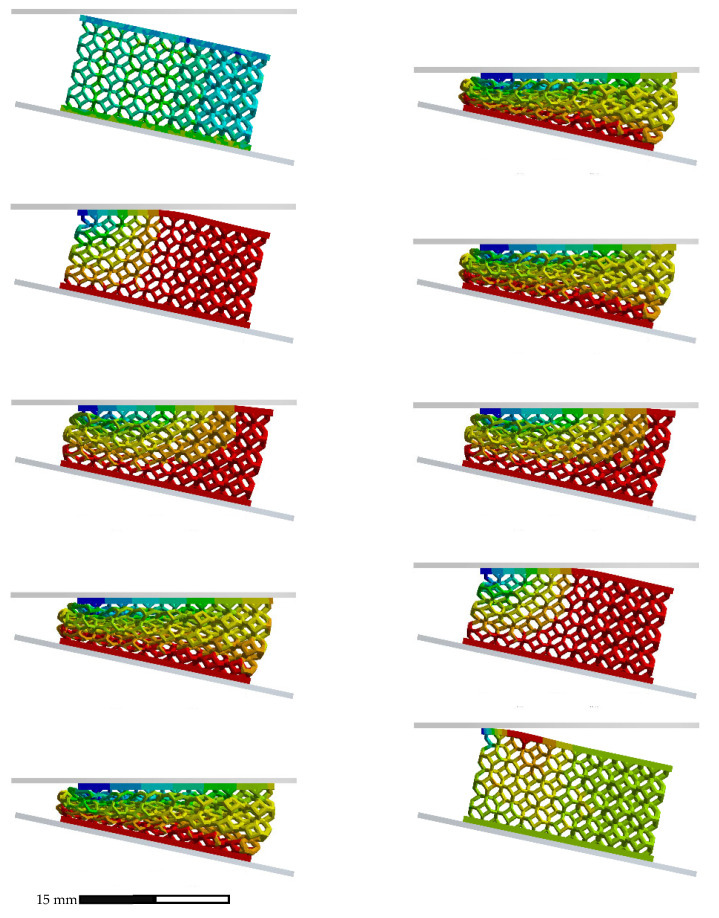
Impact sequence for side-graded lattice.

Figure 12 shows the experimental and numerical force and displacement profiles of the side-graded lattices, as well as their matching. Figure 13 displays the compression sequence of this geometry. The force-time plots of the side-graded lattice (Figure 12a) showed some similarities, but the curves did not show exactly the same pattern. The experimental maximum force of 476 N was reached around 11 ms, while the numerical maximum force of 440 N happened around 20 ms. In contrast, Figure 12b shows a better agreement with only a 9% difference in the maximum displacement, with 67 ms as the contact time for the numerical results and around 60 ms for the experimental results. The force-displacement plots (Figure 12c) show the numerical results, which had a very feeble oscillation at the beginning of the contact but that were not as intense as in the experimental results. In this case, by integrating both curves, the energy absorbed at the maximum compression differed by only 3%. The inversed side-graded results had similar behavior to the side-graded case, but the peak values were smaller, with a 392 N of maximum force and a contact time of 39 ms.

**Figure 14:**
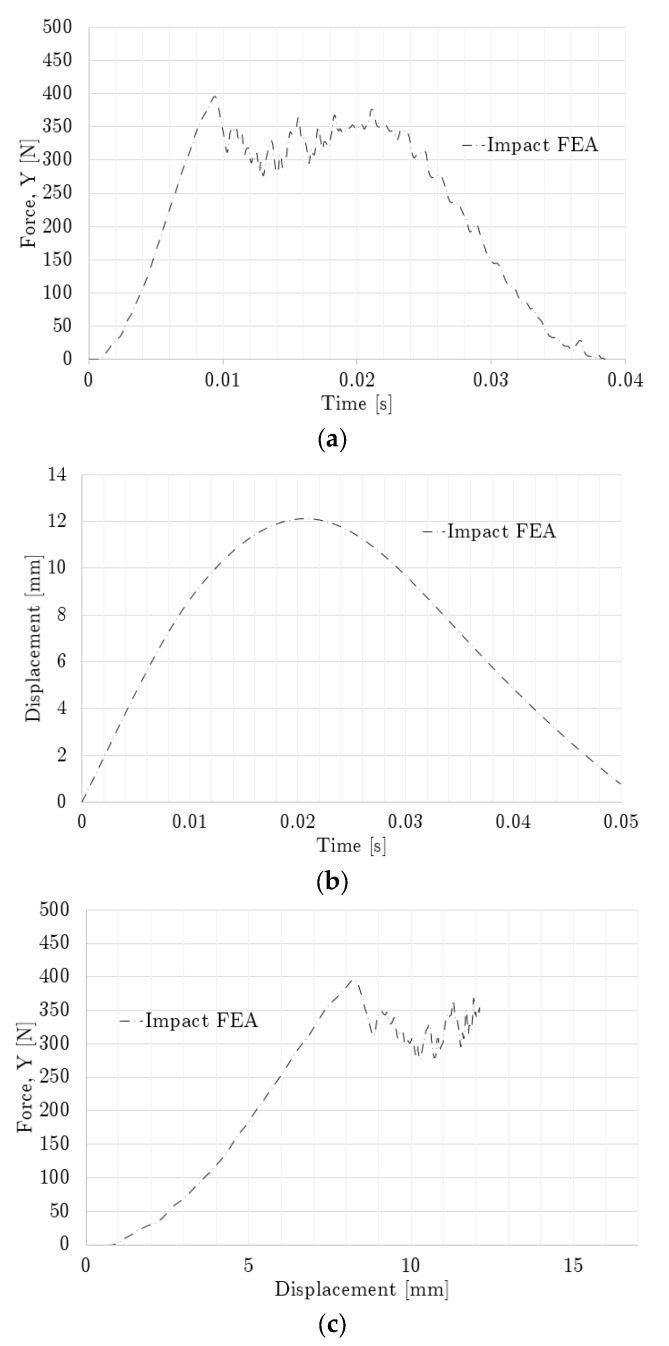
Impact loading for the inversed side-graded setup: (**a**) Force history, (**b**) Displacement history, and (**c**) Force–displacement curve.

Similar to the non-graded oblique impact case, the results for the inversed side-graded lattice were only numerical and are shown in Figure 14.

**Figure 15:**
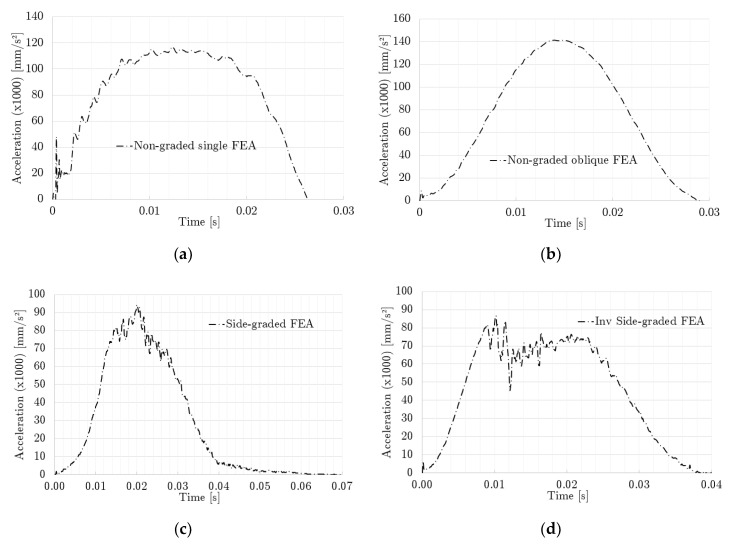
Acceleration history: (**a**) None-graded lattice single impact, (**b**) Non-graded lattice oblique impact, (**c**) Side-graded lattice, and (**d**) Inversed side-graded lattice.

Figure 15 presents the acceleration histories extracted from the impact simulation on each investigated case. By using Equation 1 the average deceleration was calculated, and the results are shown in Table 3. The average decelerations in Table 3 indicate that the side-graded lattice had a better performance since it induced less damage on the impactor than any of the other modeled cases investigated.

## 4. Conclusions

Quasi-static and impact loading tests were performed on 3D-printed TPU tensile test samples and lattices with different configurations and are shown herein. The numerical models of the tests developed in FEA commercial software were presented and discussed.

The Ogden 3rd-order hyperelastic material model proved a suitable option to model the behavior of a TPU flexible material, where only uniaxial tensile data are available. The parameter sets for this material model were obtained and reported for the quasi-static and dynamic loading cases.

The quasi-static and impact loading FEA results for the non-graded lattice showed a good agreement with the experimental data, which validates the modeling methodology and material assumptions. Thus, a similar approach can be followed in order to study other configurations and perform future analyses, such as the optimization of lattices.

The side-graded lattice FEA results resembled the experimental data, although the force results did not completely fitted; however, the displacement and contact time showed good agreement. This led us to assume with confidence that the differences observed for the side-graded lattice may be attributed to other effects not considered herein, such as geometrical differences between the lattice model and test sample, perhaps local struts fractures and/or non-uniform transversal sections.

Once validated, the numerical simulations through FEA provided information that was no available from the experiments, as was the case with the average deceleration. Based on the modeling, it was concluded that the side-graded lattice performed better that the non-graded lattice, and the additional two special cases here considered. Therefore, a similar modeling procedure can be used to address other applications, such as cushioning and impact protection, as well as evaluating additional spatially graded lattice configurations.

## Figures and Tables

**Table 1:** Ogden 3rd-order fitted parameters.

Parameter	Value	Unit
λ_1_	2.502	MPa
α_1_	−0.463	-
λ_2_	−14.542	MPa
α_2_	−2.237	-
λ_3_	−158.713	MPa
α_3_	0.103	-

**Table 2:** Ogden 3rd-order factored parameters.

Parameter	Quasi-Static Loading	Impact Loading	Unit
	Value	Value	
λ_1_	1.904	3.929	MPa
α_1_	−0.151	0.113	-
λ_2_	−12.342	−11.520	MPa
α_2_	−2.060	−2.236	-
λ_3_	189.499	−101.145	MPa
α_3_	−0.080	0.144	-

**Table 3:** Average deceleration.

Case	Average Deceleration [×1000 m/s^2^]
Non-graded simple impact	82.7
Non-graded oblique impact	74.1
Side-graded	28.0
Inversed side-graded	35.6

## Data Availability

The data presented in this study are available on request from the corresponding author.
